# Supplementary material for: Riluzole Reverses Blood–Testis Barrier Loss to Rescue Chemotherapy–Induced Male Infertility by Binding to TRPC
Source: Cells. 2024 Dec 6;13(23):2016. doi: 10.3390/cells13232016 (PMC11640501; doi:10.3390/cells13232016)
Supplement: Supplementary file 1 [file cells-13-02016-s001.zip › cells-3311716-supplementary.pdf]

1. Supplementary Figures

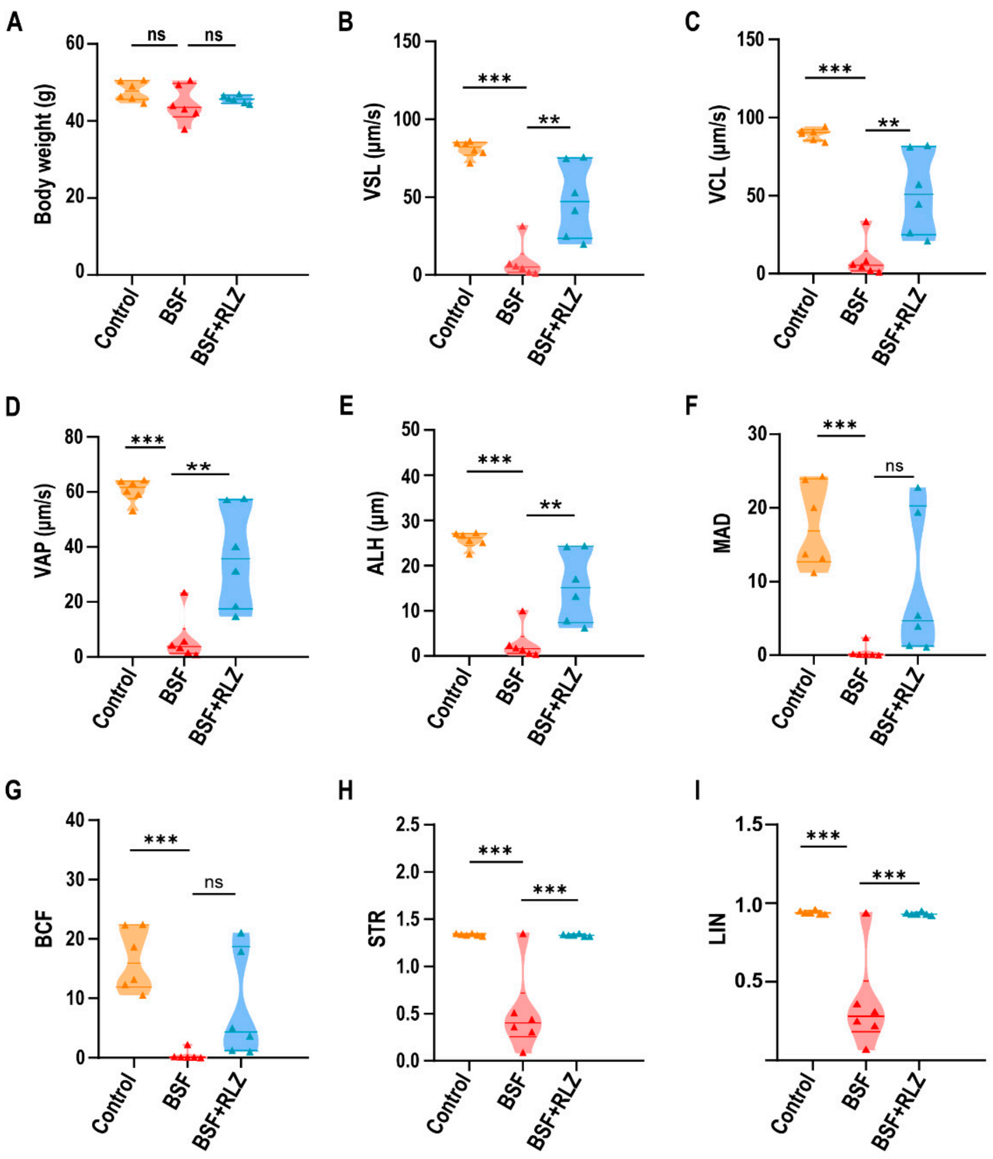

Figure S1. Riluzole administration enhanced sperm quality of oligospermic mice, related to Figure 1. (**A**) Body weight at week 8 in mice with oligospermia after riluzole treatment (n = 6). (**B-I**) Straight-line velocity (VSL), Curvilinear velocity (VCL), Average path velocity (VAP), Amplitude of lateral head displacement (ALH), Mean angular displacement (MAD), Beat-cross frequency (BCF), Straightness coefficient (STR), and Linearity (LIN) of sperms in oligospermic mice after administration of

riluzole (n=6). The data were analyzed for more sperm counts over 1000 using CASA. Values are here expressed as mean ± SEM. ***P < 0.001, **P < 0.01, *P <0.05 compared to BSF.

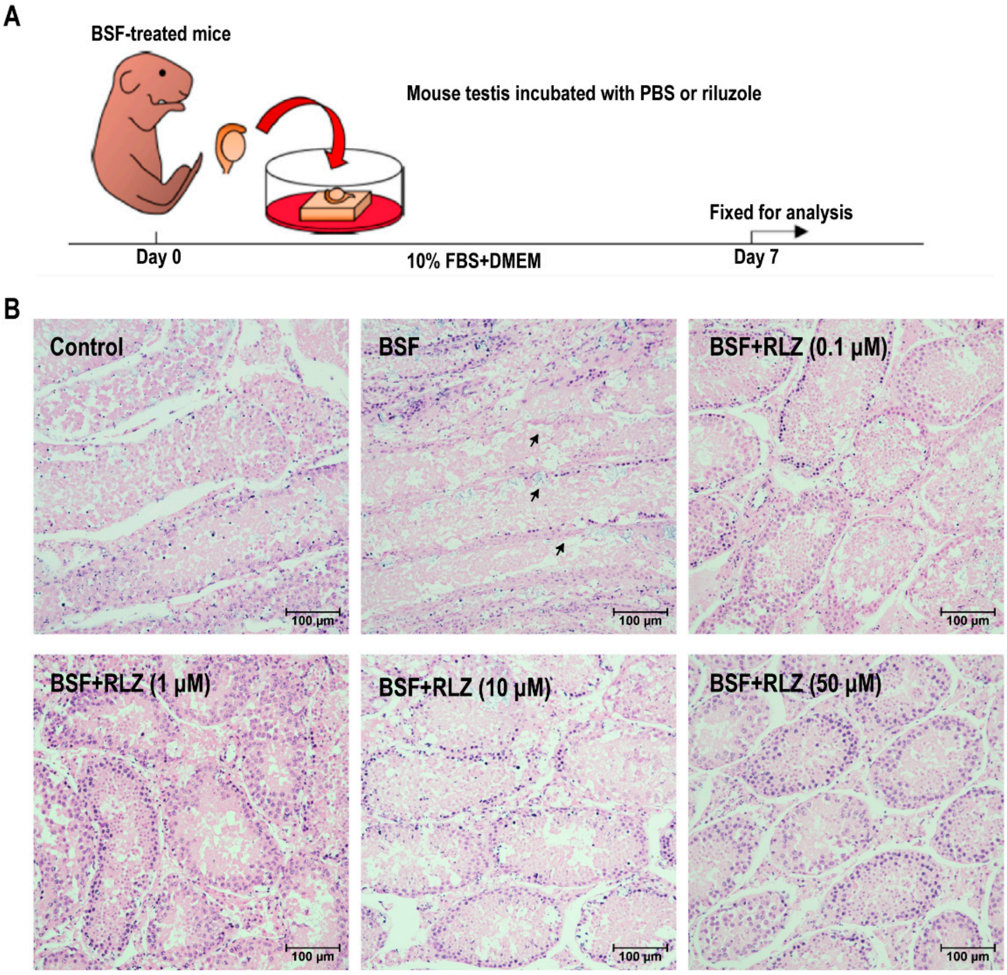

Figure S2. Validation of testicular tissue culture model in vitro. (**A**) Schematic diagram of in vitro culture of oligospermia mice testis tissue. Busulfan-induced oligospermic mice were established in 4-week-old male mice, followed by euthanization in a benchtop and transfer of testes to 1.5% agarose gel blocks in 12-well plates. Complete medium containing different concentrations of riluzole (0.1 μM, 1 μM, 10 μM, and 50

μM) was added to half the height of the agarose gel blocks. The medium was changed every two days during the culture. 7 days after administration, the testes were fixed in 4% paraformaldehyde for preparation of paraffin sections and HE staining. (**B**) Histopathological analysis of testicular tissue in vitro. The black arrow represents low numbers of germ cells in the seminiferous tubule lumen. Scale bar, 100 μm.

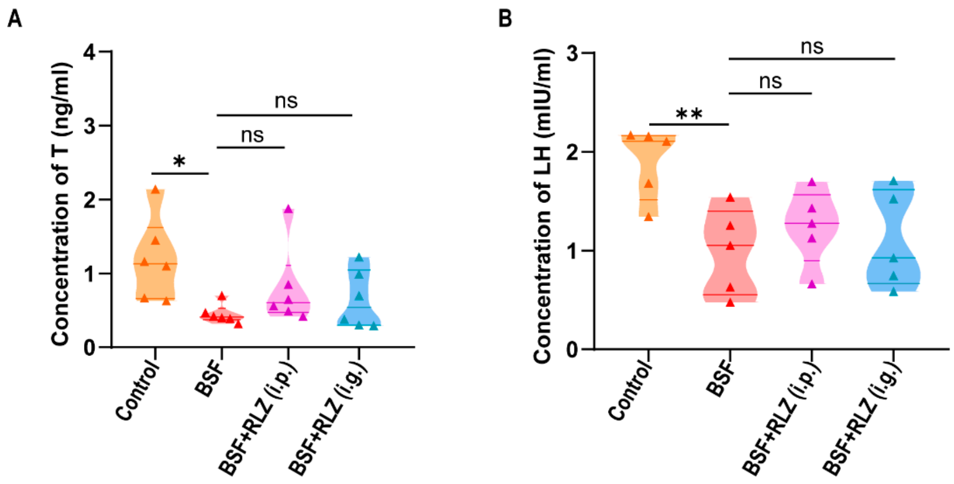

Figure S3. Riluzole had no effect on sexual hormone levels of oligospermia mice. (**A**) Testosterone concentration in serum of oligospermia mice after riluzole treatment (n=6). (**B**) LH concentration in serum of oligospermia mice after riluzole treatment (n=5). Values are here expressed as mean ± SEM. ***P < 0.001, **P < 0.01, *P <0.05 compared to BSF.

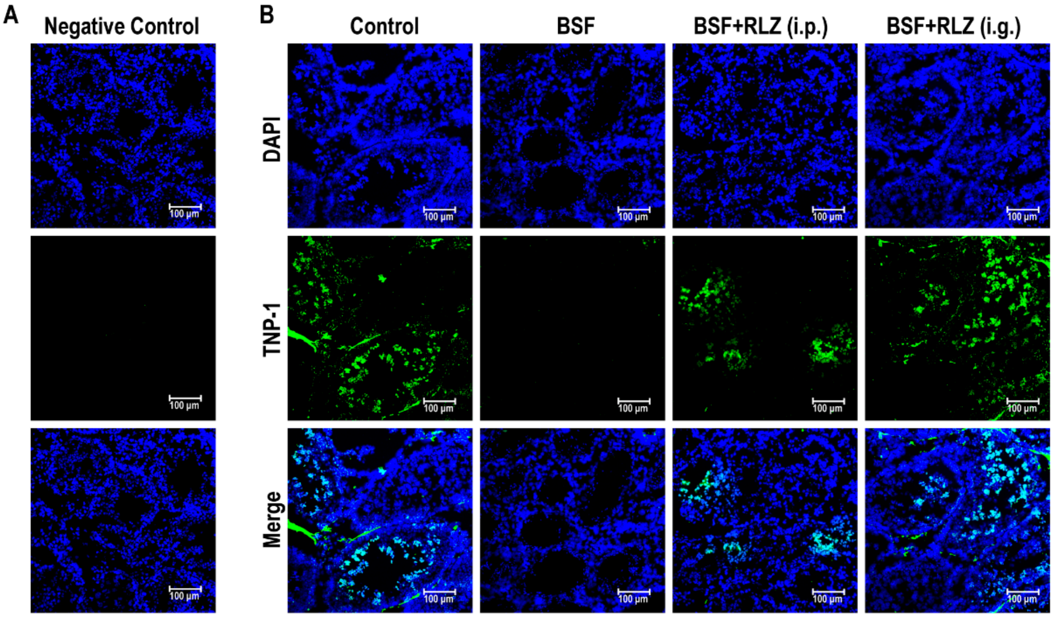

Figure S4. Effect of riluzole on spermatid marker TNP1, related to Figure 3. **(A)** Immunofluorescence staining without primary antibody was performed as a negative control. **(B)** Immunofluorescence staining showed the expression of TNP-1 in the testes of BSF-induced oligospermic mice after riluzole treatment. Cell nuclei were stained with DAPI (blue). Scale bar, 100 μm.

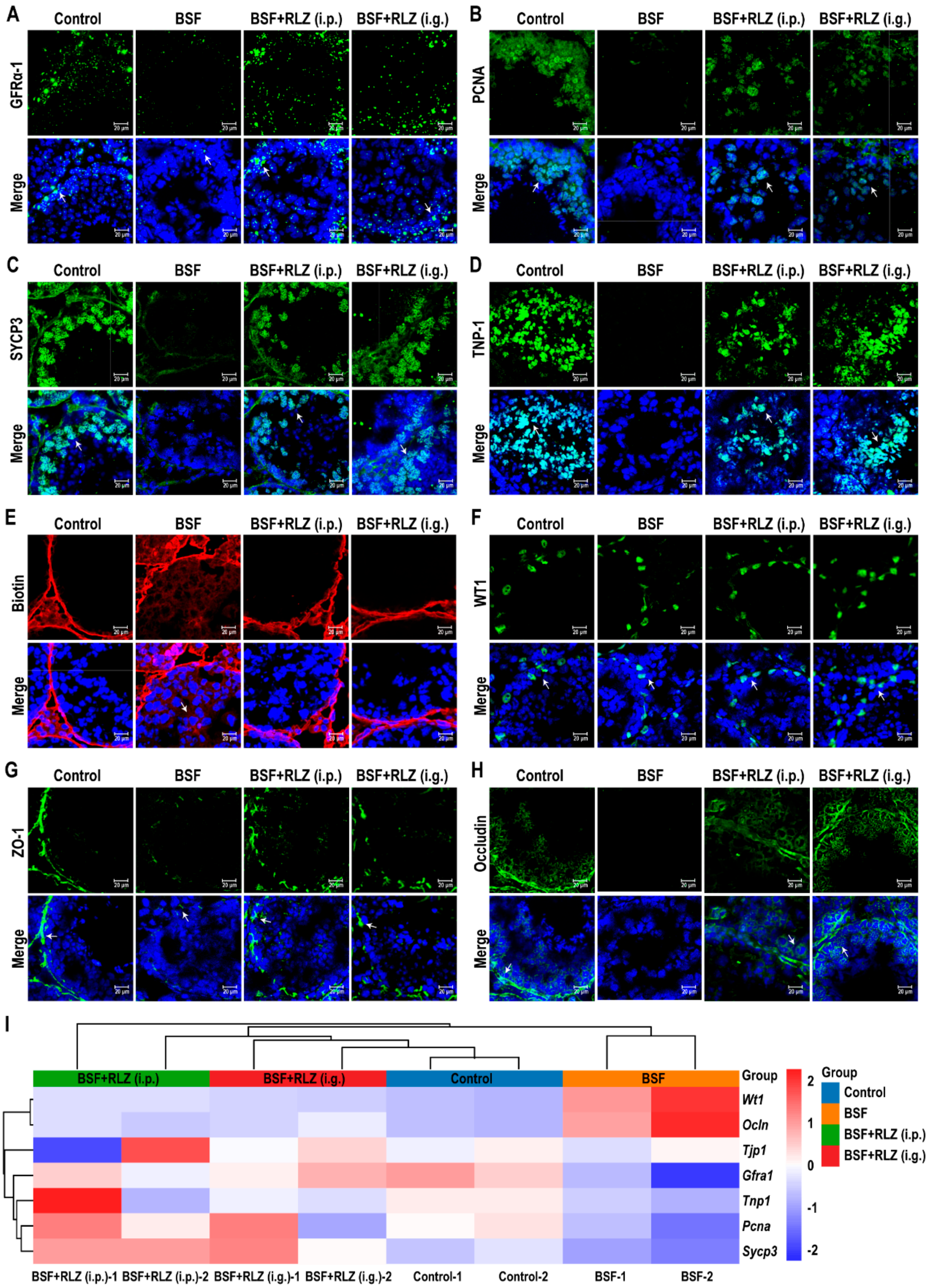

Figure S5. Effects of riluzole on key markers for proliferation and differentiation of spermatogonia and BTB integrity, related to Figure 3. **(A-H)** The higher magnification images of the pictures in Figure 3. The white arrows point to the stained cells. Scale bar,

20 µm. **(I)** The heatmap shows the changes in gene expression of *Gfra1*, *Pcna*, *Sycp3*, *Tnp1*, *Wt1*, *Tjp1,* and *Ocln*. Red and blue indicate up-and down-regulated genes, respectively.

2. Supplementary Tables

Table S1. Riluzole administration in oligospermia mice, related to Figures 1 and 2.

Group	i.p. injection of BSF (30 mg/kg·bw)	Treatment	Number
Control	None	PBS	6
BSF	Yes	PBS	6
BSF+RLZ (i.p.)	Yes	Riluzole (3 mg/kg·bw, Intraperitoneal injection)	6
BSF+RLZ (i.g.)	Yes	Riluzole (5 mg/kg·bw, Intragastric gavage)	6

Table S2. Primary antibodies used in the experiments.

Antibodies	Source	Identifier
Rabbit monoclonal to ZO-1	Abcam	Cat# ab221547; RRID: AB_2892660
Rabbit polyclonal to GFR α-1	Sigma	Cat# SAB4501166; RRID: AB_10745815
Mouse monoclonal to SCP3	Abcam	Cat# ab97672; RRID: AB_10678841
Mouse monoclonal to PCNA	Abcam	Cat# ab29; RRID: AB_303394
Rabbit polyclonal to TNP1	Abcam	Cat# ab73135; RRID:

		AB_10714560
Rabbit monoclonal to Wilms Tumor Protein	Abcam	Cat# ab89901; RRID: AB_2043201
Rabbit monoclonal to Occludin	Abcam	Cat# ab216327; RRID: AB_2737295
Connexin 43 Rabbit mAb	CST	Cat# 3512S; RRID: AB_1007667
Rabbit polyclonal to ZO1	Abcam	Cat# ab96587; RRID: AB_1007667
Rabbit polyclonal to TRPC5	Proteintech	Cat# 25890-1-AP; RRID: AB_2880285

Table S3. Primer sequences used in qRT-PCR analysis.

**Gene**	**Forward primer**	**Reverse primer**
Gdnf	CCAGTGACTCCAATATGCCTG	CTCTGCGACCTTTCCCTCTG
Bmp4	GAGCCATTCCGTAGTGCCAT	ACGACCATCAGCATTCGGTT
Scf	TGCATGGAAGAAAACGCACC	CTTTGCGGCTTTCCCTTTCTC
Cxcl12	GGTGCTCAAACCTGACGGTA	GGCAGCTCCTCTTTGGCTTA
Inhibin B	TTGCAGGTCTACGTGTGTCC	GTTTCGCCTAGTGTGGGTCA
Fgf2	TGGGTTCGAGGAAGGACTCT	AATGGTGTGGGAATGCAGGA
β-actin	CAGCCTTCCTTCTTGGGTAT	TGGCATAGAGGTCTTTACGG
